# Photodynamic Therapeutic Effect during 5-Aminolevulinic Acid-Mediated Photodynamic Diagnosis-Assisted Transurethral Resection of Bladder Tumors

**DOI:** 10.1155/2024/7548001

**Published:** 2024-07-20

**Authors:** Nobutaka Nishimura, Makito Miyake, Sayuri Onishi, Tomomi Fujii, Tatsuki Miyamoto, Mitsuru Tomizawa, Takuto Shimizu, Yosuke Morizawa, Shunta Hori, Daisuke Gotoh, Yasushi Nakai, Kazumasa Torimoto, Nobumichi Tanaka, Kiyohide Fujimoto

**Affiliations:** ^1^ Department of Urology Nara Medical University, 840 Shijo-cho, Kashihara, Nara 634-8522, Japan; ^2^ Department of Diagnostic Pathology Nara Medical University, 840 Shijo-cho, Kashihara, Nara 634-8522, Japan; ^3^ Department of Prostate Brachytherapy Nara Medical University, 840 Shijo-cho, Kashihara, Nara 634-8522, Japan

## Abstract

**Background:**

Photodynamic diagnosis-assisted transurethral resection of bladder tumors (PDD-TURBT) enhances detection of elusive lesions compared to standard white light-transurethral resection of bladder tumors (WL-TURBT). If minimal light exposure during PDD-TURBT induces the accumulation of reactive oxygen species (ROS), potentially resulting in phototoxicity in small lesions, apoptosis may be triggered in residual small tumors, allowing them to escape resection. We investigated the hypothesis of a potential photodynamic therapeutic effect during PDD-TURBT.

**Methods and Materials:**

Our study, conducted between January 2016 and December 2020 at Nara Medical University Hospital, focused on a specific emphasis on ROS production. Immunohistochemical analysis for thymidine glycol and N^*ε*^-hexanoyl-lysine was performed on 69 patients who underwent 5-aminolevulinic acid-mediated PDD-TURBT and 28 patients who underwent WL-TURBT. Additionally, we incrementally applied the minimal irradiation energy to T24 and UM-UC-3 cells treated with 5-aminolevulinic acid using instruments similar to those used in PDD-TURBT and evaluated intracellular ROS production and phototoxicity.

**Results:**

Immunohistochemical analysis revealed a significant increase in production of thymidine glycol and N^*ε*^-hexanoyl-lysine within the PDD-TURBT group. In T24 and UM-UC-3 cells treated with 5-aminolevulinic acid and light exposure, immunofluorescent staining demonstrated a dose-dependent increase in intracellular ROS production. In addition, higher irradiation energy levels were associated with a greater increase in ROS production and phototoxicity, as well as more significant decrease in mitochondrial membrane potential.

**Conclusion:**

Although the irradiation energy used in PDD-TURBT did not reach the levels commonly used in photodynamic therapy, our findings support the presence of a potential cytotoxic effect on bladder lesions during PDD-TURBT.

## 1. Introduction

Transurethral resection of bladder tumors (TURBT) is the primary diagnostic and therapeutic approach for nonmuscle invasive bladder cancer (NMIBC). Recently, photodynamic diagnosis (PDD)-assisted TURBT has garnered attention owing to its ability to enhance tumor detection rates and reduce intravesical recurrence [1–3]. PDD is a medical imaging technique used to detect cancerous tissues, particularly in the bladder during TURBT procedures. The term “photodynamic” comes from the Greek words “photo,” meaning light, and “dynamics,” meaning force or power. In PDD, a photosensitizing agent, such as 5-aminolevulinic acid (5-ALA) or hexaminolevulinate (HAL), is administered to the patient. These agents preferentially accumulate in cancerous tissues. When the bladder is illuminated with blue light during cystoscopy, the cancerous tissues fluoresce (emit a red or pink light), making them more visible compared to normal tissues. This enhanced visibility aids surgeons in identifying and removing cancerous tissues more accurately [4, 5]. The effectiveness of PDD-TURBT in preventing intravesical recurrence is primarily attributed to its superior ability to detect microlesions that are challenging to identify with conventional white light-transurethral resection of bladder tumors (WL-TURBT) [6–9].

In cancer cells, 5-aminolevulinic acid (5-ALA) is not metabolized into heme in the mitochondria, leading to the intracellular accumulation of the intermediate metabolite protoporphyrin IX (PpIX) [9]. PpIX is a photosensitizer that is activated and emits red fluorescence (around 635 nm) when exposed to blue light (400−410 nm) provided by a laser. Additionally, in photodynamic therapy (PDT), red light with wavelengths ranging from 600 to 800 nm is commonly used, leading to the production of reactive oxygen species (ROS) by PpIX [10–12]. Red light is frequently used in PDT due to its deeper tissue penetration capabilities and its effectiveness in activating photosensitizers that generate cytotoxic ROS, which are crucial for the treatment's efficacy. On the other hand, in PDD-TURBT, the purpose is the detection of tumors. Tumors with high accumulation of PpIX are exposed to blue light, causing them to emit red fluorescent light. This is why blue light is used during PDD-TURBT. Tumor observation typically begins under blue light, but after circumferential marking around tumor, the procedure usually switches to white light mode for tumor resection. White light has a broad spectrum, encompassing wavelengths around 600−800 nm commonly used in PDT. We believe that the significant decrease in intravesical recurrence rate of PDD-TURBT is attributed to the efficient mechanical resection of tumors facilitated by the visualization of tumors through red fluorescence and this approach helps prevent any residual tumor during PDD-TURBT. Additionally, we hypothesized that PDD-TURBT could potentially induce PDD effect in very small and flat lesions that may be difficult to detect even with PDD-TURBT. Many clinical trials of PDT for bladder cancer have utilized a relatively high energy dose of 25–100 J/cm^2^ when applying PDT with 5-ALA [13–15]. This energy dose was considerably higher than that emitted by the light source used for TURBT. However, we consider the possibility that during PDD-TURBT, minimal light exposure induces the accumulation of ROS similar to PDT, resulting in phototoxicity in small tumors that are difficult to detect even with PDD-TURBT. Compared to PDT, the energy levels of PDD-TURBT are obviously lower. They might be too low to induce apoptosis in bladder cancer. However, what if a PDT-like effect occurs even at the low energy levels of PDD-TURBT? The residual tumors from resection might be caused to die because of this PDT-like effect, which could potentially contribute to a decrease in intravesical recurrence. Here, we conducted a series of experiments to investigate whether these effects similar to PDT are achieved in tumor tissue and tumor cells with the minimal energy generated during PDD-TURBT.

## 2. Materials and Methods

### 2.1. Cell Culture

In order to translate this experiment to actual clinical settings, we used two bladder urothelial cancer cell lines, namely T24 and University of Michigan-Urinary Carcinoma-3 (UM-UC-3). These cells are derived from human bladder cancer. For example, T24 is derived from muscle-invasive bladder cancer, and UM-UC-3 is derived from NMIBC. The cell lines were maintained in RPMI1640 medium (Nacalai Tesque, Kyoto, Japan) supplemented with 10% fetal bovine serum (Nichirei Biosciences Inc., Tokyo, Japan), 100 U/mL penicillin and 100 µg/mL streptomycin (Nacalai Tesque, Kyoto, Japan) in a standard humidified incubator at 37°C in an atmosphere containing 5% CO_2_.

### 2.2. Tumor Specimens Obtained by TURBT

All procedures involving human participants performed in this study were in accordance with the ethical standards of the Nara Medical University Ethical Committee (Ethical Approval Number: 1256) and the 1964 Helsinki Declaration and its later amendments or comparable ethical standards.

**Figure 1:**
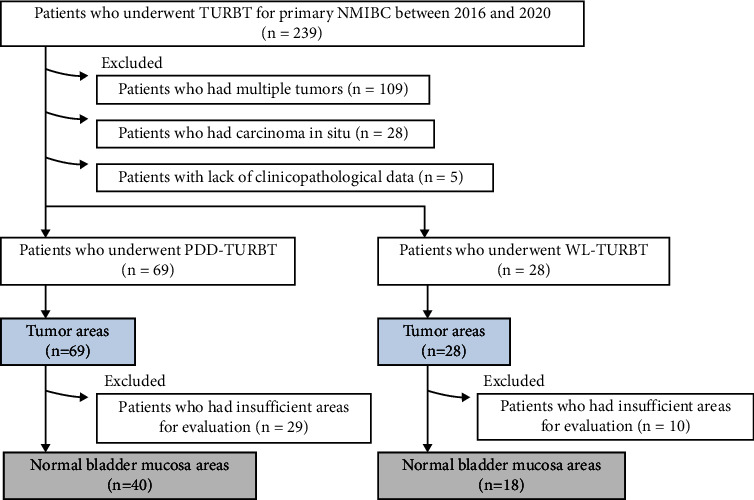
Flowchart of patient selection for immunohistochemical staining analysis. Of the 239 patients who underwent TURBT for primary NMIBC between 2016 and 2020, 69 who underwent PDD-TURBT and 28 who underwent WL-TURBT were included in this study. NMIBC, nonmuscle invasive bladder cancer; PDD, photodynamic diagnosis; TURBT, transurethral resection of bladder cancer; WL, white light.

Immunohistochemical staining was performed to evaluate ROS production in tumor tissues during transurethral surgery. A total of 239 patients with primary NMIBC underwent initial WL or PDD TURBT between January 2016 and December 2020 at Nara Medical University Hospital. Of these, 109 patients who had multiple tumors, 28 patients who had carcinoma *in situ*, and five patients with a lack of clinicopathological data were excluded from this study, leaving 69 patients who underwent PDD-TURBT and 28 patients who underwent WL-TURBT (Figure 1). To establish the control group for the TURBT group, tumor areas and normal mucosa areas were extracted from the specimens of both groups. Patients with sufficient tumor area for evaluation were included in this analysis. The following section describes how we evaluated tumor and normal bladder mucosa areas for immunohistochemical analysis. For the immunohistochemical analysis, tumor areas from all 69 patients in the PDD-TURBT group and 28 patients in the WL-TURBT group were used. However, for the immunohistochemical analysis of the bladder mucosa area, 29 patients in the PDD-TURBT group and 10 patients in the WL-TURBT group were excluded from this analysis because their specimens did not have sufficient volume of bladder mucosa. Finally, 69 tumor areas and 40 normal bladder mucosa areas in the PDD-TURBT group were included in this study. Additionally, 28 tumor areas and 18 normal bladder mucosa areas in the WL-TURBT group were included in this study.

### 2.3. Immunohistochemical Staining Analysis

Immunohistochemical staining was performed as previously described [16]. Briefly, antibodies against thymidine glycol (TG) and N^*ε*^-hexanoyl-lysine (N^*ε*^-HEL) were used as primary antibodies (dilution ratio of 1 : 100 for overnight incubation at 4°C) and were purchased from the Japan Institute for the Control of Aging, NIKKEN SEIL Co, Ltd (Shizuoka, Japan). The secondary antibody, peroxidase-conjugated anti-mouse immunoglobulin G (H + L) (Nichirei Biosciences Inc., Tokyo, Japan), was used at a dilution ratio of 1 : 1,000.

**Figure 2:**
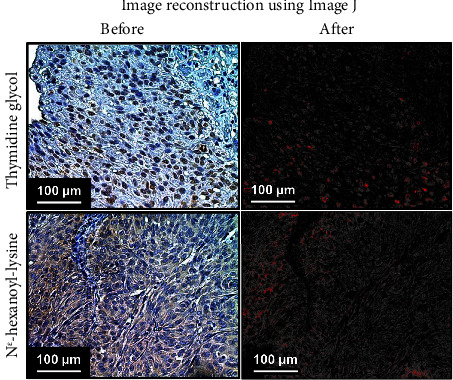
Micrographs before and after using ImageJ to reconstruct immunohistochemical staining analysis. The areas stained through immunohistochemical staining analysis were reconstructed as red using ImageJ, allowing the calculation of the percentage of stained area per field of view.

ImageJ (National Institute of Health, Rockville, MD, USA, freeware) with Java™ version 1.8.0_112 (64-bit) under Windows 10 Pro edition was used to quantify stain-positive cells [17].  Figure 2 shows representative images of immunohistochemical staining analysis using anti-TG and N^*ε*^-HEL primary antibodies, as well as images reconstructed using ImageJ. For immunohistochemical staining analysis, five sites were randomly selected from each specimen and observed under a high-power field, and the percentage of stained areas in one field of view was measured using ImageJ. Furthermore, we evaluated ROS production in the normal bladder mucosa area as well as in tumors from PDD-TURBT. The percentage of the area stained with TG and N^*ε*^-HEL was measured by dividing the staining area of TG and N^*ε*^-HEL by the total tumor area and total bladder mucosa area in one field of view, respectively. For each specimen, the mean stained area was calculated from five fields of view and the mean values were compared between the PDD and WL-TURBT groups.

### 2.4. PDT Using 5-ALA and WL Exposure to Urothelial Cancer Cells

Experimental PDT procedures were performed in dark conditions as much as possible. The cells were initially seeded at a density of 0.5 × 10^5^ cells per well in 24-well plates. Subsequently, the cells were treated with and without 1 mM 5-ALA. The previous study reported that higher ALA concentrations were associated with increased PpIX accumulation in urothelial cell lines, with a defined concentration of 1 mM for 5-ALA [18]. The cells treated with 5-ALA were kept in the dark for 3 hours within a standard humidified incubator at 37°C with 5% CO_2_, followed by WL exposure at increasing intensities of 0.01, 0.02, 0.05, 0.1, 0.2, 0.5, and 1.0 mJ/cm^2^ using a TURBT light source (KARL STORZ GmbH & Co. KG; Tuttlingen, Germany). During WL exposure, the cumulative energy was measured using an irradiance meter (Delta OHM, Padova, Italy). Supplementary Figure S1 shows a photograph taken during WL exposure. The cells treated with PDT were incubated for an additional 1 hr within a standard humidified incubator at 37°C with 5% CO_2_.

### 2.5. Immunofluorescent Staining Analysis

Immunofluorescent staining of the cultured cells was performed as previously described [19]. Cells treated with PDT were fixed with 4% paraformaldehyde, permeabilized with phosphate-buffered saline (PBS) containing 0.1% Triton X-100 (PBS-T) (Nacalai Tesque, Kyoto, Japan), and blocked with PBS-T containing 1% bovine serum albumin (BSA). The primary anti-TG and N^*ε*^-HEL antibodies were added to the samples at a dilution ratio of 1 : 100 in PBS-T containing 1% BSA overnight at 4°C in a humidified chamber. Subsequently, a goat anti-mouse IgG (H + L) highly cross-adsorbed secondary antibody (Thermo Fischer Scientific, Massachusetts, US) at a 1 : 500 dilution in PBS-T containing 1% BSA was added to the samples at room temperature for 1 h in the dark. Nuclear counterstaining and mounting were performed simultaneously using Ibidi Mounting Medium with DAPI (NIPPON Genetics Co., Ltd. Japan).

For immunofluorescent staining, the mean percentage of primary antibody-positive cells among the DAPI-stained cells was compared for each irradiation energy level. Cell counting was performed automatically using ImageJ software (National Institute of Health, Rockville, MD, USA, freeware), similar to the immunohistochemical staining analysis. The phototoxicity induced by PDT was evaluated by the WST-8 assay using a Cell Counting Kit-8 (Dojindo Molecular Technologies, Kumamoto, Japan). 100 *μ*L of WST-8 solution was added to each well, and the cells were further incubated at 37°C for 1 h. Absorbance was measured at 450 nm using a microplate reader (TECAN, Männedorf, Switzerland). For statistical comparisons, three wells were prepared for each condition.

### 2.6. Measurement of ROS Production

The accumulation of ROS from the experimental PDT procedures was assessed using the Highly Sensitive DCFH-DA-ROS assay kit (Dojindo Molecular Technologies, Kumamoto, Japan). DCFH-DA is a dye used to detect ROS with high sensitivity. Briefly, the cells were washed twice with hypo- and normo-VICs using Hanks' Balanced Salt Solution (HBSS) (FUJIFILM Wako Chemicals Co., Japan) and then treated with the highly sensitive DCFH-DA dye working solution for 30 minutes. Fluorescence intensity was measured using a microplate reader (TECAN, Männedorf, Switzerland). Fluorescence intensity measurements were conducted using an excitation wavelength of 490 nm and an emission wavelength of 540 nm. For statistical comparisons, three wells were prepared for each condition.

### 2.7. Measurement of Mitochondrial Membrane Potential

Mitochondrial membrane potential (MMP) was evaluated using the JC-1 MitoMP Detection Kit (Dojindo Molecular Technologies, Kumamoto, Japan). Mitochondria are also the primary site of ROS production within cells. In the ATP production pathway via oxidative phosphorylation, leaked electrons react with oxygen, producing superoxide anions, which are highly reactive ROS. Additionally, mitochondrial DNA and the electron transport chain are susceptible to damage by ROS. With aging, mitochondrial dysfunction occurs, leading to a decrease in mitochondrial membrane potential. This kit allows for the evaluation of mitochondrial membrane potential and assessment of the potential for cell apoptosis. JC-1 emits red fluorescence when it aggregates in mitochondria with normal membrane potential. When the membrane potential decreases, JC-1 exists as a monomer and emits green fluorescence. Changes in the intensity of red and green fluorescence can be used to assess the state of the mitochondria. Normally, JC-1 emits both green and red fluorescence, but as the membrane potential decreases, the red fluorescence diminishes and the green fluorescence becomes more intense. After the experimental PDT procedures, cells were exposed to JC-1 solution in the culture medium at a final concentration of 4 *μ*M and then incubated at 37°C for 30 minutes. After two washes with culture medium, fluorescence intensity was measured using a microplate reader (TECAN, Männedorf, Switzerland). The fluorescence ratio (Red/Green) was calculated and compared between two groups treated with and without PDT. For green fluorescence intensity, measurements were conducted with an excitation wavelength of 485 nm and an emission wavelength of 530 nm. For red fluorescence intensity, measurements were conducted with an excitation wavelength of 535 nm and an emission wavelength of 600 nm. For statistical comparisons, three wells were prepared for each condition.

### 2.8. Statistical Analysis

Statistical analyses for immunohistochemical staining, immunofluorescent staining, the WST-8 assay, the ROS assay, and the mitochondrial membrane potentials were conducted using the Mann–Whitney *U* test. Statistical analyses of patient characteristics were conducted using appropriate tests, such as Student's *t*-test and the chi-square test. Box plots were constructed using the GraphPad Prism 7.0 software (GraphPad Software, San Diego, CA, USA). Statistical significance was set at a *P* value <0.05. These analyses were performed using EZR software (Saitama Medical Center, Jichi Medical University, Saitama, Japan) [20].

## 3. Results

### 3.1. Production of TG and N^*ε*^-HEL during PDD-TURBT and WL-TURBT

**Figure 3:**
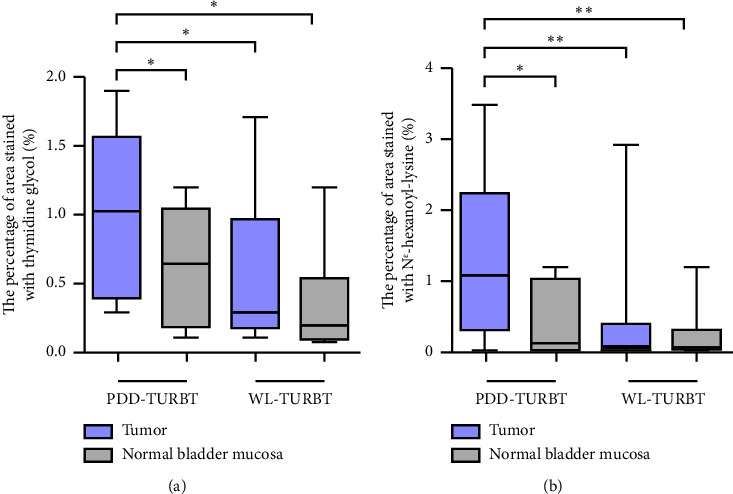
A comparison of the percentage of area stained with TG and N^*ε*^-HEL in immunohistochemical staining analysis. Box plots show the comparison of area stained with TG (a) and N^*ε*^-HEL (b) between PDD- and WL-TURBT groups that, respectively, included patients who underwent initial PDD- and WL-TURBT for primary NMIBC. The percentage of area stained with TG and N^*ε*^-HEL was measured by dividing the staining area of TG and N^*ε*^-HEL by the total tumor area and total bladder mucosa area in one field of view, respectively. The asterisk (^*∗*^) indicates a *P* value <0.05, and double asterisks (^*∗∗*^) indicate a *P* value <0.01. PDD, photodynamic diagnosis; TURBT, transurethral resection of bladder cancer; WL, white light.

Table 1 lists the clinicopathological characteristics of the patients and compares the PDD and WL-TURBT groups. No significant differences were observed in age, sex, tumor size, clinical T category, grade, lymphovascular invasion, and variant histology. Additionally, the average time between sample collection by TURBT and evaluation of immunohistochemical staining analysis was 23.9 months (standard deviation [SD], ±11.2) in the PDD-TURBT group and 44.8 months (SD, ±11.3) in the WL-TURBT group (*P* < 0.001). The average surgical time was 22.5 minutes (SD, ±7.6) for the PDD-TURBT group and 20.9 minutes (SD, ±7.5) for the WL-TURBT group.  Figure 3 shows a comparison of area positive for TG and N^*ε*^-HEL between the PDD- and WL-TURBT groups. TG is expressed in the nucleus when thymidine is damaged by hydroxyl radicals, and it is used to evaluate DNA damage [21]. N^*ε*^-HEL is a marker of lipid peroxidation-induced oxidative stress [22]. The production of TG and N^*ε*^-HEL in cancer cells was significantly higher in the PDD-TURBT group compared to the WL-TURBT group. Additionally, ROS production in the PDD-TURBT-treated tumor group was significantly higher than in the normal bladder mucosa group.

### 3.2. PDT-Induced Intracellular Production of TG and N^*ε*^-HEL in T24 and UM-UC-3

**Figure 4:**
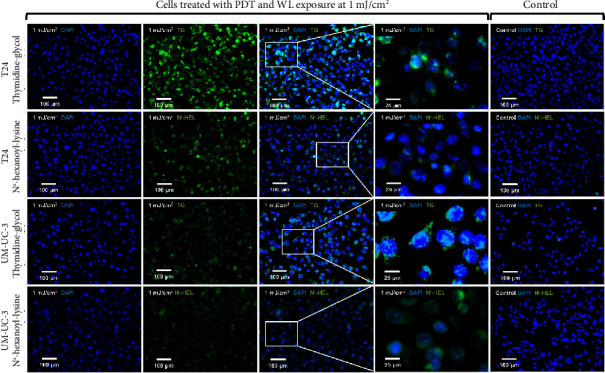
Micrographs showing immunofluorescent staining analysis using TG and N^*ε*^-HEL expression. Micrographs of T24 and UM-UC-3 cells treated with PDT at 1 mJ/cm^2^ WL exposure are shown. These cancer cells were stained with TG and N^*ε*^-HEL in immunofluorescent staining analysis. Immunofluorescent staining requires two days, so all images were taken the day after PDT. TG and HEL reflect oxidation of nucleic acids and lipid due to ROS, respectively. The significant increase in green fluorescence compared to the control after 1 mJ/cm^2^ of WL exposure suggests an increase in the production of these oxidative stress markers. In the control group, cells were not exposed to 5-ALA and were treated with PDD-TURBT. N^*ε*^-HEL, N^*ε*^-hexanoyl-lysine; PDT, photodynamic therapy; TG, thymidine glycol: WL, white light.

**Figure 5:**
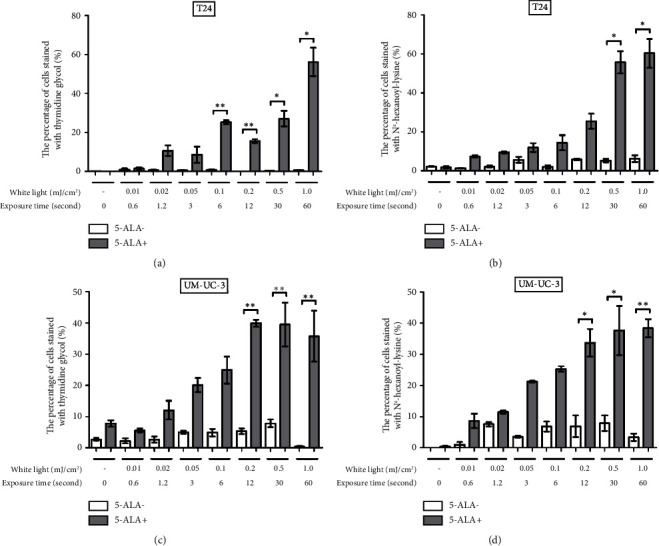
Evaluation of percentage of cells stained for TG and N^*ε*^-HEL at each irradiation energy level. T24 (a, b) and UM-UC-3 (c, d) cells were treated with or without 5-ALA and WL exposure at increasing intensity of 0.01, 0.02, 0.05, 0.1, 0.2, 0.5, and 1.0 mJ/cm^2^. The exposure time for each energy level is also indicated. The percentage of cells stained for TG and N^*ε*^-HEL is compared between the cells with and without the experimental PDT procedures at each irradiation energy level. Since the staining process takes two days, this analysis was immediately conducted on the day following WL exposure. For statistical comparisons, three wells were prepared for each condition. An asterisk (^*∗*^) indicates a *P* value <0.05, and double asterisks (^*∗∗*^) indicate a *P* value <0.01. 5-ALA, 5-aminolevulinic acid; WL, white light.

Figure 4 shows representative images of immunofluorescent staining analysis of T24 and UM-UC-3 cells treated with PDT and 1.0 mJ/cm^2^ of WL exposure. In both T24 and UM-UC-3 cells, more than half of the cells were positive for the production of TG and N^*ε*^-HEL.  Figure 5 shows a comparison of cells positive for TG and N^*ε*^-HEL at each irradiation energy level that were treated with and without PDT. Approximately 0.6 seconds of exposure resulted in an irradiation energy level of 0.01 mJ/cm^2^, and with 1 minute of exposure, it reached exactly 1.0 mJ/cm^2^. In both T24 and UM-UC-3 cells, only a few cells were positive for TG and N^*ε*^-HEL at an irradiation energy level of 0.01 and 0.02 mJ/cm^2^. Immunofluorescent staining demonstrated that the production of intracellular ROS increased in a dose-dependent manner with increasing light exposure level. Furthermore, at 0.5 and 1.0 mJ/cm^2^, the percentage of stained cells for TG and N^*ε*^-HEL were significantly higher in the PDT-treated cells.

### 3.3. PDT-Induced Phototoxicity

**Figure 6:**
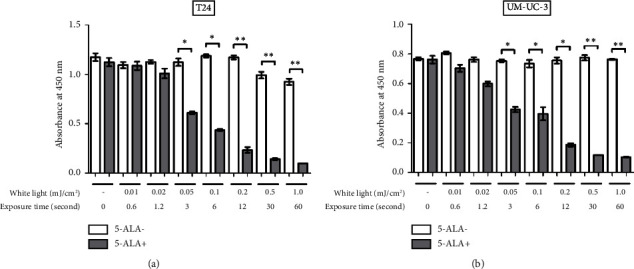
Evaluation of phototoxicity at each irradiation energy level. The phototoxicity induced by PDT was evaluated by the WST-8 assay using a Cell Counting Kit-8 (Dojindo Molecular Technologies, Kumamoto, Japan). The lower absorbance at 450 nm indicates higher phototoxicity. This analysis was conducted immediately following WL exposure. T24 (a) and UM-UC-3 (b) cells were treated with or without 5-ALA and exposed to WL at increasing intensities of 0.01, 0.02, 0.05, 0.1, 0.2, 0.5, and 1.0 mJ/cm^2^. The absorbance is compared between the cells with and without the experimental PDT procedures at each irradiation energy level. The exposure time for each energy level is also indicated. For statistical comparisons, three wells were prepared for each condition. An asterisk (^*∗*^) indicates a *P* value <0.05, and double asterisks (^*∗∗*^) indicate a *P* value <0.01. 5-ALA, 5-aminolevulinic acid; WL, white light.

Figure 6 shows a comparison between T24 and UM-UC-3 cells treated with and without PDT for phototoxicity at each irradiation energy level. PDT-induced phototoxicity increased in a dose-dependent manner with increasing light exposure level. Significant PDT-induced toxicity was observed in T24 and UM-UC-3 cells treated with PDT using 0.05 mJ/cm^2^ or more.

### 3.4. PDT-Induced ROS Production

**Figure 7:**
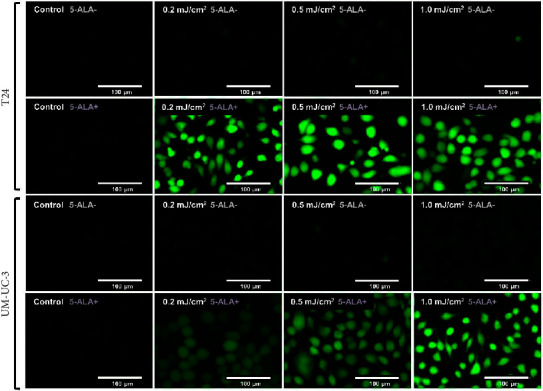
Micrographs showing PDT-induced intracellular ROS production. Micrographs show PDT-induced intracellular ROS production in T24 and UM-UC-3 cells treated with or without 5-ALA at 0.2, 0.5, and 1.0 mJ/cm^2^ of WL exposure. This analysis was conducted using the highly sensitive DCFH-DA-ROS assay kit (Dojindo Molecular Technologies, Kumamoto, Japan), and these images were taken immediately after WL exposure. The green fluorescence reflects the intracellular accumulation of ROS. 5-ALA, 5-aminolevulinic acid.

**Figure 8:**
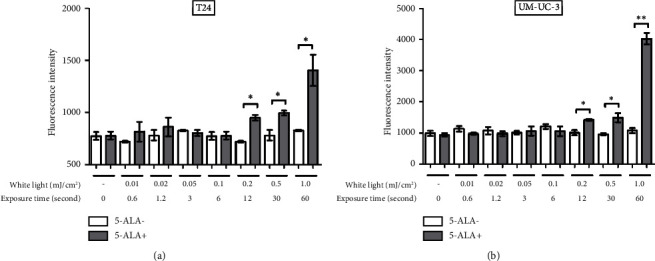
Evaluation of intracellular ROS production at each irradiation energy level. PDT-induced ROS production was evaluated using the highly sensitive DCFH-DA-ROS assay kit (Dojindo Molecular Technologies, Kumamoto, Japan). This analysis was conducted immediately following WL exposure. T24 (a) and UM-UC-3 (b) cells were treated with or without 5-ALA and exposed to WL at increasing intensities of 0.01, 0.02, 0.05, 0.1, 0.2, 0.5, and 1.0 mJ/cm^2^. The fluorescence intensity was compared between cells with and without the experimental PDT procedures at each irradiation energy level. The higher fluorescence intensity indicates an increase in ROS production. For statistical comparisons, three wells were prepared for each condition. An asterisk (^*∗*^) indicates a *P* value <0.05, and double asterisks (^*∗∗*^) indicate a *P* value <0.01. 5-ALA, 5-aminolevulinic acid.

Figure 7 shows representative images of fluorescence micrographs of ROS production in T24 and UM-UC-3 cells treated with and without PDT, and 0.2, 0.5, and 1.0 mJ/cm^2^ of WL exposure. In both T24 and UMUC3 cells, we observed minimal intracellular ROS production in the cells without PDT at any irradiation energy level. However, in the cells treated with PDT, increasing fluorescence intensity correlating with higher energy levels was observed.  Figure 8 shows a comparison of fluorescence intensity between the cells treated with and without PDT at each irradiation energy level. In both T24 and UMUC3 cells, a significant increase in ROS production was observed in cells treated with PDT compared to those without PDT, starting from an energy level of 0.2 mJ/cm^2^, which corresponds to an irradiation time exceeding 12 seconds.

### 3.5. Changes in Mitochondrial Membrane Potential Induced by PDT

**Figure 9:**
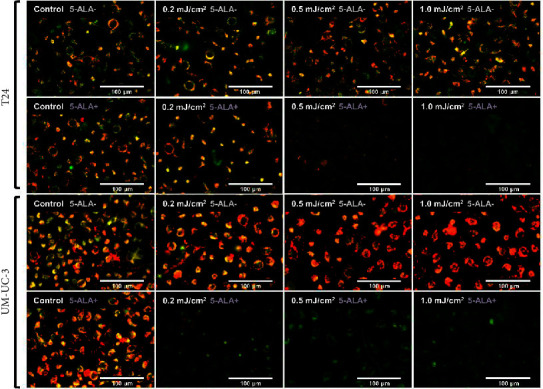
Micrographs showing changes of mitochondrial membrane potential induced by PDT. Micrographs show changes of mitochondrial membrane potential induced by PDT in T24 and UM-UC-3 cells treated with or without 5-ALA at 0.2, 0.5, and 1.0 mJ/cm^2^ WL exposure. These images were taken using the JC-1 MitoMP detection kit (Dojindo Molecular Technologies, Kumamoto, Japan) immediately after the experimental PDT procedures. This kit allows the evaluation of mitochondrial membrane potential and the assessment of the potential for cell apoptosis. JC-1 emits red fluorescence when it aggregates in mitochondria with normal membrane potential. When the membrane potential decreases, JC-1 exists as a monomer and emits green fluorescence. The changes in the intensity of red and green fluorescence can be used to assess the state of the mitochondria. Normally, JC-1 emits both green and red fluorescence, but when the membrane potential decreases, the red fluorescence diminishes and the green fluorescence becomes more intense. Eventually, the red fluorescence represents mitochondrial membrane potential. A decrease in red fluorescence indicates a reduction in mitochondrial membrane potential, which could induce apoptosis. 5-ALA, 5-aminolevulinic acid.

**Figure 10:**
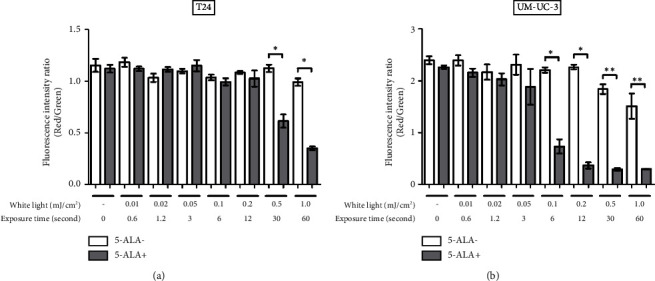
Evaluation of changes of mitochondrial membrane potential induced by PDT. Changes in mitochondrial membrane potential induced by PDT were evaluated using the JC-1 MitoMP Detection Kit (Dojindo Molecular Technologies, Kumamoto, Japan) immediately following WL exposure. T24 (a) and UM-UC-3 (b) cells were treated with or without 5-ALA and exposed to WL at increasing intensities of 0.01, 0.02, 0.05, 0.1, 0.2, 0.5, and 1.0 mJ/cm^2^. The fluorescence intensity ratio (red/green) was compared between cells with and without the experimental PDT procedures at each irradiation energy level. The lower fluorescence intensity ratio indicates a reduction of mitochondrial membrane potential. For statistical comparisons, three wells were prepared for each condition. An asterisk (^*∗*^) indicates a *P* value <0.05, and double asterisks (^*∗∗*^) indicate a *P* value <0.01.

Figure 9 shows representative images of fluorescence micrographs of mitochondrial membrane potential in T24 and UM-UC-3 cells treated with PDT and 0.2, 0.5, and 1.0 mJ/cm^2^ of WL exposure. Mitochondrial membrane potential is indicated by red fluorescence. There is no reduction in red fluorescence in cells without PDT at any irradiation energy level. However, in cells treated with PDT, a decrease in red fluorescence is observed with increasing irradiation energy.  Figure 10 shows a comparison of fluorescence intensity ratio (Red/Green) between cells treated with and without PDT at each irradiation energy level. In T24 cells, a decrease in mitochondrial membrane potential was observed when the irradiation energy exceeded 0.5 mJ/cm^2^. Similarly, in UM-UC-3 cells, a decrease in mitochondrial membrane potential was observed when the irradiation energy exceeded 0.1 mJ/cm^2^.

## 4. Discussion

Several studies have investigated the *in vivo* dynamics of ROS production. Generally, ROS can induce cell death in tumor cells, making them potential candidates for disease treatment [23]. 5-ALA-PDT relies on a combination of visible light exposure and PpIX generation within cells. Exposure to light in the presence of PpIX and molecular oxygen leads to the formation of cytotoxic intermediates that induce tumor cell death [23]. Although this effect has been utilized in applications such as brain tumors, esophageal cancer, and skin cancer, 5-ALA-PDT has not been widely adopted for bladder cancer.

Szliszka et al. reported the potential of combined therapy using tumor necrosis factor-related apoptosis-inducing ligand (TRAIL) and PDT to induce cytotoxicity in bladder cancer cells [24]. TRAIL can selectively induce apoptosis in cancer cells; however, T24 cells are resistant to TRAIL and showed enhanced cytotoxicity in combination with 5-ALA-PDT. In this report, visible light with a wavelength of 400–750 nm was used and an irradiation energy level was 7.5 J/cm^2^. Ekroll et al. reported on the cell destruction mechanism induced by PDT using the rat bladder cancer cell line AY-27, with a dose of 1.6 J/cm^2^ [25]. Numerous studies have been conducted on this topic. The irradiation energy used in PDD-TURBT does not reach the levels used in PDT, which requires 20–100 J/cm^2^ to achieve adequate cytotoxic effects on bladder cancer [26, 27]. Moreover, the wavelengths used in PDD-TURBT and PDT are different. PDD-TURBT uses blue light (410–420 nm), whereas PDT uses higher wavelengths (650–660 nm). The depth of laser penetration differs as well. Although we cannot directly compare the energy levels between PDD-TURBT and PDT, it is clear that the energy level of PDD-TURBT is significantly lower than that of PDT. This likely results in different levels of ROS production. In PDT, the high level of ROS production may contribute to a more efficient cancer cell death. The ROS induced by PDT might also affect other lesions in combination with systemic therapies such as chemotherapy and immune checkpoint inhibitors [28]. In contrast, PDD-TURBT likely does not have such effects due to its lower ROS production.

In PDT, we cannot ignore the adverse events, including bladder spasms, hematuria, and urinary urgency [29, 30]. These adverse events are caused by the high level of ROS production. However, in the potential PDT effect introduced by PDD-TURBT, these adverse events do not occur. In fact, symptoms such as urinary urgency and hematuria after PDD-TURBT are caused by the resection of the bladder mucosa, not the PDT effect. Therefore, we can suggest that the potential PDT effect by PDD-TURBT might be obviously safer.

The phototoxicity of PDD-TURBT was confirmed in our study. Furthermore, using the TURBT device, we observed accumulation of ROS within the bladder cancer cells and confirmed a decrease in mitochondrial membrane potential when the irradiation time exceeded approximately 10 seconds. This result suggests that during actual PDD-TURBT procedures, even with minimal irradiation time, there is a possibility of inducing phototoxicity in tumor cells located on the bladder mucosal surface. However, all experiments in this study were conducted in vitro, lacking structures such as microvasculature and stroma present in actual tumor tissues, which could not be taken into consideration. Drawing conclusions solely from these results can be challenging; however, using the PDD-TURBT device, we were able to confirm the accumulation of ROS within mitochondria and phototoxicity with very low doses of WL exposure.

We compared oxidative stress markers, including TG and N^*ε*^-HEL, using specimens obtained from TURBT. When evaluating ROS production, we must consider the influence not only of PDT-like effects but also of the surgical procedure itself, which can induce ROS production through physical stimuli. To address this, we divided patients into two groups: PDD-TURBT and WL-TURBT. By ensuring both groups experienced similar levels of surgical invasion from physical stimuli, we conducted statistically appropriate analyses to mitigate concerns about the surgical procedure's influence. Ultimately, our findings suggest that PDD-TURBT may introduce higher ROS production compared to WL-TURBT. In an actual bladder cancer scenario, cancer cells are densely layered, and it is unlikely that the light from PDD-TURBT with PpIX would reach the deep cancer cells. Similarly, red light commonly used in PDT with PpIX is mostly at 630 nm that is believed to penetrate to a depth of approximately 5 mm [31]. Under white light exposure, tissue penetration depth is lower. Furthermore, laser light sources are specifically manufactured for PDT, primarily using slender fibers to efficiently deliver light to the tumor, and their energy efficiency is not comparable to the light irradiation used in TURBT [32]. The radiance meter for TURBT light source showed an irradiation energy level of 1.0 mJ/cm^2^ per 60 seconds. Even if it were to be applied continuously for one hour, it would only reach a cumulative value of 60 mJ/cm^2^. In reality, even with continuous irradiation for one hour, such a simple calculation may not apply because, during PDD-TURBT, the endoscope is constantly moved within the bladder, so it never remains in one place for long. However, it may be effective against residual tumors and very small lesions after TURBT. If there is a photodynamic therapeutic effect with PDD-TURBT, it may be beneficial to take the time during surgery to slowly and carefully observe while applying light to the entire bladder mucosa. For example, during PDD-TURBT, the operation period is sufficiently long and the entire bladder mucosa is exposed to light from the TURBT instrument, potentially reducing the intravesical recurrence rate. This optimistic effect is attributed not only to the high detection rate resulting from thorough observation of the bladder mucosa but also to the potential PDT effect.

This study has several limitations. First, experiments on ROS production and phototoxicity following light irradiation were conducted exclusively using cultured cells. This study was conducted entirely in vitro, which limits reproducibility. Actual tumors are thicker, posing challenges due to the limited tissue penetration depth of white light used in TURBT. Unfortunately, this aspect was not investigated in our study. Ideally, future research would involve a 3D cell model or in vivo studies using nude mice to address these limitations. Second, the evaluation of ROS production and phototoxicity was only based on immunostaining using oxidative stress markers, the WST-8 assay, the Highly Sensitive DCFH-DA-ROS assay kit, and the JC-1 MitoMP Detection Kit. Singlet oxygen used in PDT can induce apoptosis through both the extrinsic and intrinsic pathways. Methods allowing a more direct measurement of ROS levels are available, including electron spin resonance spectroscopy and the direct measurement of mitochondrial ROS [33]. Therefore, alternative approaches should have been explored in addition to this study. Specifically, multiplex staining would be suitable for assessing multiple indices simultaneously. Unfortunately, we are currently unable to utilize such a technique. Third, there was a significant delay between sample collection by TURBT and evaluation of immunohistochemical staining analysis. This duration is approximately 2 years for the PDD-TURBT group and 4 years for the WL-TURBT group. ROS have a short half-life and are not expected to persist in the samples. We relied on oxidative stress markers in the cells resulting from ROS, which are known to remain for a certain period even after formalin fixation. However, the exact duration of retention is not definitive, and in cases where samples have aged for several years, conducting an accurate evaluation may not be feasible. Fourth, the number of TURBT samples used for immunohistochemistry was limited. Excluding more than half of the initially included patients who underwent TURBT for primary NMIBC between 2016 and 2020 introduced significant selection bias. Consequently, the cohort decreased from 239 to 97, leading to decreased reproducibility. Therefore, further accumulation of bladder cancer cases is necessary.

## Figures and Tables

**Table 1:** Clinicopathological characteristics between PDD- and WL-TURBT groups.

Variables	PDD-TURBT group *n* (%)	WL-TURBT group *n* (%)	*P* value
*N*	69 (71.3)	28 (28.7)	
Time between TURBT and immunohistochemical staining analysis, months, mean ± SD	23.9 ± 11.2	44.8 ± 11.3	<0.001
Surgery time, minutes, mean ± SD	22.5 ± 7.6	20.9 ± 7.5	0.359
Age at initial TURBT, years, mean ± SD	75.6 ± 8.4	74.7 ± 9.2	0.668
Sex			0.512
Male	61 (88.4)	23 (82.1)	
Female	8 (11.6)	5 (17.9)	
Tumor size			0.427
<30 mm	55 (79.7)	20 (71.4)	
≥30 mm	14 (20.3)	8 (28.6)	
Clinical T category			0.627
Ta	47 (68.1)	21 (75.0)	
T1	22 (31.9)	7 (25.0)	
Grade (WHO1973)			0.181
Grade 1	2 (2.9)	1 (3.6)	
Grade 2	40 (58.0)	21 (75.0)	
Grade 3	19 (27.5)	6 (21.4)	
Unknown	8 (11.6)	0 (0.0)	
Grade (WHO2004)			1.000
Low grade	48 (69.6)	20 (71.4)	
High grade	21 (30.4)	8 (28.6)	
LVI			0.199
Negative	68 (98.6)	26 (92.9)	
Positive	1 (1.4)	1 (3.6)	
Variant histology			1.000
Negative	69 (100.0)	28 (100.0)	
Positive	0 (0.0)	0 (0.0)	

LVI, lymphovascular invasion; PDD, photodynamic diagnosis; SD, standard deviation; TURBT, transurethral resection of bladder tumor; WHO, World Health Organization; WL, white light.

## Data Availability

The datasets generated and/or analyzed during the current study are available from the corresponding author on reasonable request.

## Abbreviations

BSA: Bovine serum albumin
HBSS: Hanks' balanced salt solution
MMP: Mitochondrial membrane potential
N^*ε*^-HEL: N^*ε*^-hexanoyl-lysine
NMIBC: Nonmuscle invasive bladder cancer
PBS: Phosphate-buffered saline
PDD-TURBT: Photodynamic diagnosis-assisted transurethral resection of bladder tumors
PDT: Photodynamic therapy
PpIX: Protoporphyrin IX
ROS: Reactive oxygen species
TURBT: Transurethral resection of bladder tumors
TG: Thymidine glycol
UM-UC-3: University of Michigan-urinary carcinoma-3
WL-TURBT: White light-transurethral resection of bladder tumors
5-ALA: 5-aminolevulinic acid.

## Supplementary Materials

Supplementary Materials Supplementary Figure S1: a photograph during WL exposure. The WL exposure was carried out using a TURBT endoscope (KARL STORZ GmbH & Co. KG, Tuttlingen, Germany) in a light-shielded 24-well plate. At the same time, the irradiance meter was used to measure the irradiation energy. All procedures were performed in the dark. TURBT, transurethral resection of bladder tumor.
