# Supplementary figures and images for: Photodynamic Therapeutic Effect during 5-Aminolevulinic Acid-Mediated Photodynamic Diagnosis-Assisted Transurethral Resection of Bladder Tumors
Source: Adv Urol. 2024 Jul 20;2024:7548001. doi: 10.1155/2024/7548001 (PMC11300098; doi:10.1155/2024/7548001)

Supplementary Figure S1


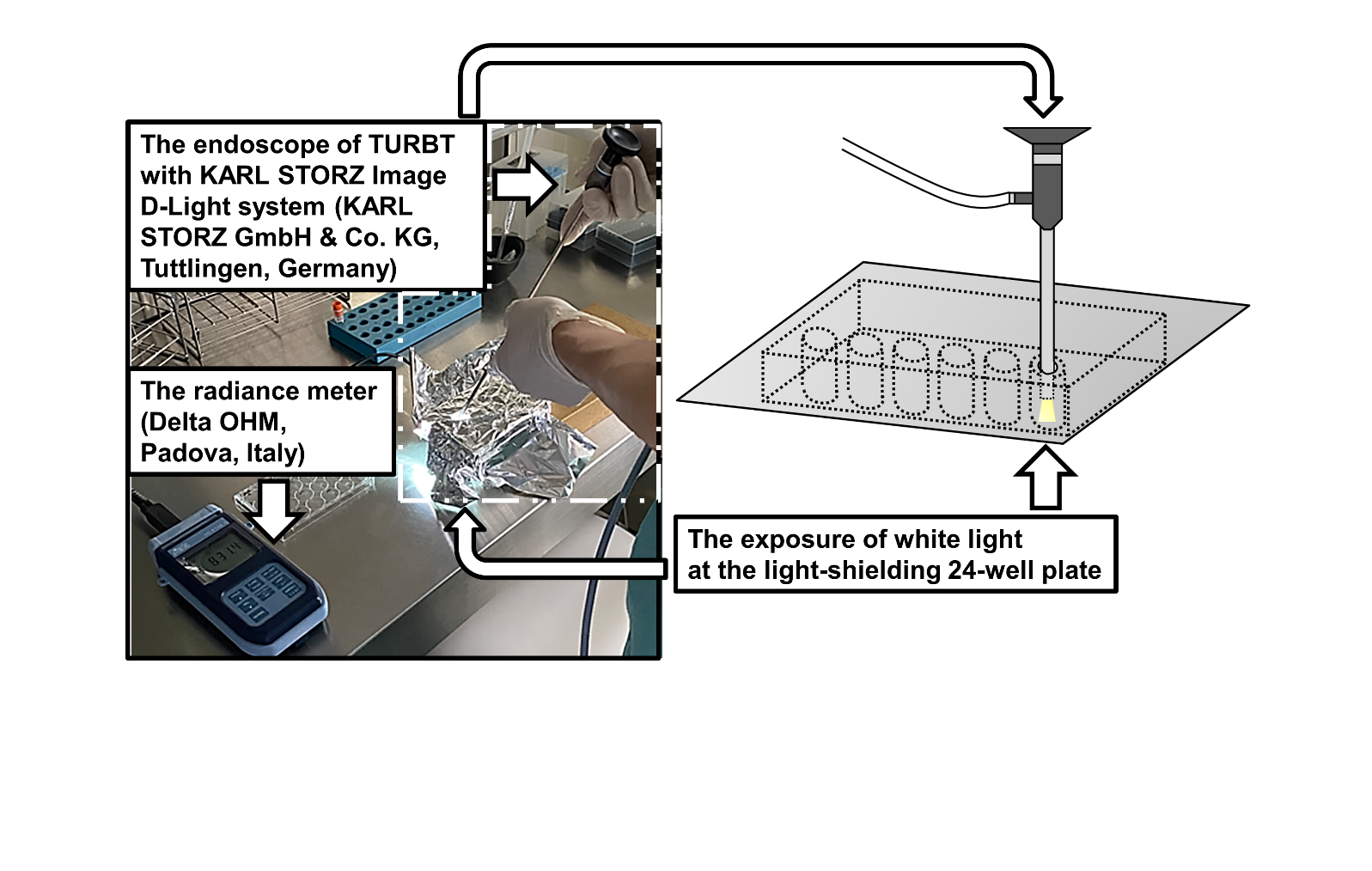

Supplement: Supplementary Materials — Supplementary Figure S1: a photograph during WL exposure. The WL exposure was carried out using a TURBT endoscope (KARL STORZ GmbH & Co. KG, Tuttlingen, Germany) in a light-shielded 24-well plate. At the same time, the irradiance meter was used to measure the irradiation energy. All procedures were performed in the dark. TURBT, transurethral resection of bladder tumor. [file 7548001.f1.docx]
